# Delineating Biophysical Environments of the Sunda Banda Seascape, Indonesia

**DOI:** 10.3390/ijerph120201069

**Published:** 2015-01-22

**Authors:** Mingshu Wang, Gabby N. Ahmadia, Iliana Chollett, Charles Huang, Helen Fox, Anton Wijonarno, Marguerite Madden

**Affiliations:** 1 Center for Geospatial Research, Department of Geography, University of Georgia, Athens, GA 30602, USA; E-Mail: mmadden@uga.edu; 2 Conservation Science Program, World Wildlife Fund—US, Washington, D.C. 20037, USA; E-Mails: Gabby.Ahmadia@gmail.com (G.N.A.); charleshuang80@gmail.com (C.H.); hfox@rare.org (H.F.); 3 Marine Spatial Ecology Lab, College of Life and Environmental Sciences, University of Exeter, Exeter, Devon EX4 4SB, UK; E-Mail: iliana.chollett@gmail.com; 4 Marine Spatial Ecology Lab, School of Biological Sciences, University of Queensland, St. Lucia Brisbane, QLD 4072, Australia; 5 Coral Triangle Program, World Wildlife Fund—Indonesia, Jakarta Selatan 12540, Indonesia; E-Mail: awijonarno@wwf.or.id

**Keywords:** biophysical environments, Sunda Banda Seascape, remote sensing, self-organizing map, sea surface temperature, chlorophyll a, currents, salinity, marine conservation

## Abstract

The Sunda Banda Seascape (SBS), located in the center of the Coral Triangle, is a global center of marine biodiversity and a conservation priority. We proposed the first biophysical environmental delineation of the SBS using globally available satellite remote sensing and model-assimilated data to categorize this area into unique and meaningful biophysical classes. Specifically, the SBS was partitioned into eight biophysical classes characterized by similar sea surface temperature, chlorophyll a concentration, currents, and salinity patterns. Areas within each class were expected to have similar habitat types and ecosystem functions. Our work supplemented prevailing global marine management schemes by focusing in on a regional scale with finer spatial resolution. It also provided a baseline for academic research, ecological assessments and will facilitate marine spatial planning and conservation activities in the area. In addition, the framework and methods of delineating biophysical environments we presented can be expanded throughout the whole Coral Triangle to support research and conservation activities in this important region.

## 1. Introduction

Marine ecosystems are constantly threatened by both natural and anthropogenic disturbances. Increasing anthropogenic pressure, such as coastal development jeopardizes resilience and resistance of the ecosystem to cope with natural disturbance and threatens the health of marine environments. More than 2.2 billion people reside within 100 km of coastlines [1], and the number of humans living near coastlines is expected to increase from 2.3 billion in 2000 to 3.1 billion in 2025 [2]. These ecosystems are critical because they support livelihoods by providing food, protecting coastlines, maintaining fisheries and sustaining tourism [3,4]. Many of these coastal inhabitants rely directly on the wealth of natural resources and services provided by marine ecosystems for subsistence and as a source of income. Compounded with the effects of climate change, marine systems require innovative strategies to managing their environment, and need to develop marine protected areas (MPA) that are resilient to both resource exploitation and the effects of climate change. Although resources need to be expended to sustain local livelihoods, coastal ecosystems need to be managed to secure their ecological function and availability of marine resources in the future.

In recent decades, oceanographic studies have described the dynamic range of environmental heterogeneity in tropical ecosystems [5,6,7,8,9]. Biophysical environments dictate the structure and function of marine ecosystems, serve as proxies for the distribution of species and habitats (which are difficult to obtain at large scales), and facilitate conservation planning. When prioritizing conservation activities on a broad spatial scale, there are often gaps of information about the ecosystems present in the area. A classification of biophysical environments using remote sensing can be a useful first step towards a comprehensive understanding of the region of focus [5,10], the identification of priority sites for rapid assessment or monitoring activities and the building of ecologically representative MPA networks that incorporate each habitat type in each of its environments.

**Figure 1:**
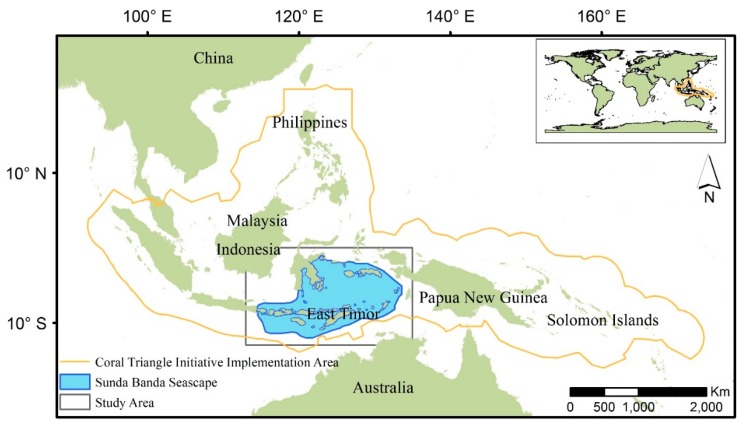
Coral Triangle and Sunda Banda Seascape. The world base map is courtesy of ESRI. The boundary of the Coral Triangle and the Sunda Banda Seascape were obtained from The Coral Triangle Atlas (ctatlas.reefbase.org/). Administrative boundaries were acquired from GADM database of Global Administrative Areas (www.gadm.org/).

The Coral Triangle (Figure 1) refers to a roughly triangular area of the tropical marine waters of Indonesia, Malaysia, Papua New Guinea, Philippines, Solomon Islands and East Timor (aka Timor-Leste) [11]. The 5.7 million km^2^ area is recognized as an epicenter of tropical marine biodiversity [12]. The ecosystem services of the Coral Triangle sustain over 120 million people, who rely on its coral reefs for food, income, and protecting coastlines from storms. Although worldwide, 60% of reefs are currently threatened by local stressors such as overfishing, destructive fishing, coastal development and pollution, this value is as high as 85% in the Coral Triangle [13]. The most widespread local threat in the region is overfishing [13]. Much of the fishing that occurs is unsustainable and jeopardizes people’s livelihoods. Moreover, a changing climate—changes in ocean chemistry, warming temperatures, increased frequency of storms—is exacerbating local anthropogenic disturbances on the Coral Triangle [14]. In order to address these urgent threats, the Coral Triangle Initiative on Coral Reefs, Fisheries and Food Security (CTI-CFF, http://www.coraltriangleinitiative.org), a multilateral partnership including six countries, was formed in 2007. The CTI-CFF has become one of the largest conservation initiatives in the marine world with considerable financial support from multilateral donors and foundations.

In the central part of the Coral Triangle in Indonesian waters lies the Sunda Band Seascape (SBS) (see Figure 1). According to the Ministry of Marine and Fisheries Affairs of Indonesia, the SBS has been designated as the second most important marine ecological region in Indonesia in terms of its biodiversity, providing habitat for 76% of known coral species and more than 3000 fish species. However, similar to the Coral Triangle as a whole, the SBS is threatened by human activities related to unsustainable development.

The SBS covers an area of almost 1.57 million km^2^ and encompasses considerable environmental and biological heterogeneity. In order to support marine conservation planning in this area, a systematic classification of the region in terms of biophysical environments is needed. Although information on marine provinces is available at a global scale [15,16], no comprehensive regional information is available for the SBS at a spatial resolution that is meaningful for management. Here, the goal of this research was to develop an approach using globally-available satellite remote sensing and model-assimilated data to categorize this area into unique and meaningful classes based on a suite of biophysical conditions. Specifically, the SBS was partitioned into biophysical classes characterized by similar sea surface temperature patterns, chlorophyll a concentration, currents, and salinity. These classes are expected to have similar habitat types and ecosystem function. Our work is the first of this kind in the SBS, and will help designate priorities in conservation planning and inform marine conservation practices.

## 2. Materials and Methods

The Sunda-Banda Seascape (SBS) study area is located in the central portion of the Coral Triangle in eastern Indonesia (Figure 1). Sunda Banda refers to a geological and geographical area of the landscape covering the marine area and islands from Bali in the southwest to Maluku in the northeast.

A number of biophysical variables were considered for characterizing the marine environment of the SBS and geospatial data sources for these variables were identified. Salinity and sea surface temperature are fundamental determinants of global distribution of many marine habitats and ecosystems [17]. In the Pacific region, ranges and extremes of sea surface temperature (SST) and chlorophyll a [18] control coral reef ecosystems. Hydrodynamic conditions, such as ocean currents, can ultimately determine both location and extent of marine habitats. For example, the distribution of mangrove ecosystems is driven by major ocean currents [19]. Therefore, biophysical variables used in this study include SST, chlorophyll a, currents and salinity.

The SST and chlorophyll a concentration data employed in this study were derived by the U.S. National Aeronautics and Space Administration (NASA) from remote sensing imagery acquired by the Moderate Resolution Imaging Spectroradiometer (MODIS) on NASA’s Aqua Satellite. MODIS mapped data (Level 3), at a spatial resolution of 4 km per pixel, were acquired over the period July 2002 to June 2013. Monthly averaged night-time SST data, chlorophyll a data, and climatological SST data were downloaded from NASA (http://oceancolor.gsfc.nasa.gov). Then, the long-term mean SST (Avg SST) was calculated. Maximum and minimum values at pixel level were selected to derive variables of highest monthly climatological SST (Max SST) and lowest monthly climatological SST (Min SST), respectively. The long-term mean chlorophyll a (Chla) concentration (mg/m^3^) was also calculated.

Daily ocean currents data from May 2008 to July 2013 were obtained from the Hybrid Coordinate Ocean Model (HYCOM, http://hycom.org/), a multi-institutional effort sponsored by the U.S. National Ocean Partnership Program [20]. We used global data-assimilative runs at 1/12° equatorial spatial resolution and 10 m depth. For this study, the long-term mean of current speed (m/s) was used. Daily salinity data at 10 m depth from May 2008 to July 2013 were also obtained from HYCOM, and the long-term mean salinity was used as input for the classification analyses.

**Figure 2:**
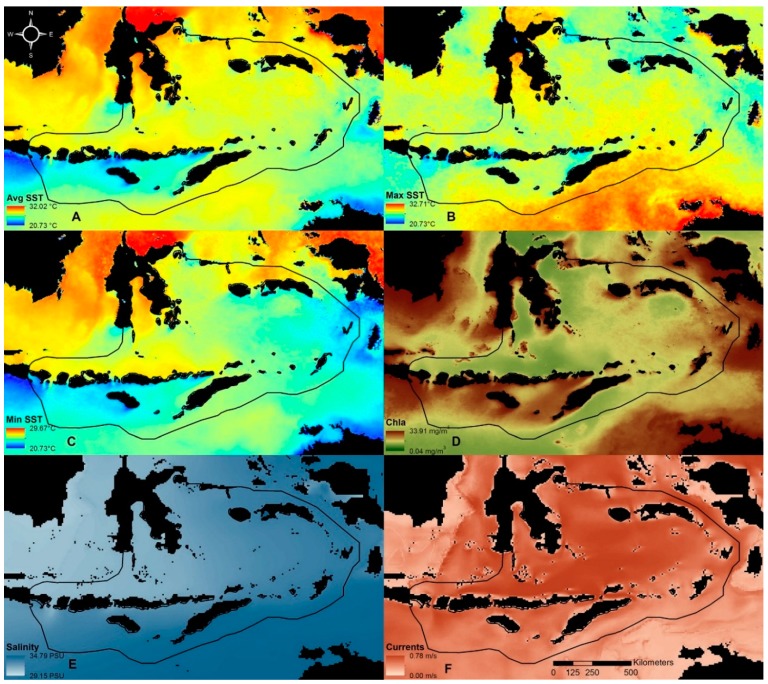
Input variables for classifying the Sunda Banda Seascape into biophysical regions. (**A**) Average sea surface temperature (Avg SST); (**B**) maximum monthly climatological sea surface temperature (Max SST); (**C**) minimum monthly climatological sea surface temperature (Min SST); (**D**) average chlorophyll a concentration (Chla); (**E**) sea salinity (Salinity); (**F**) average ocean current speed (Currents).

Data processing included the retrieval and cropping of the global datasets into the region of interest (113°E to 135°E and 0°to 13°S). HYCOM derived currents and salinity data were resampled into 4 km spatial resolution using bicubic interpolation in order to match MODIS derived datasets of Avg SST, Max SST, Min SST and Chla (Figure 2). Compared to other interpolation methods (e.g., nearest neighbor and bilinear interpolation), bicubic interpolation usually gives smother results. Land masses were identified using MODIS data and were excluded from the analysis (shown as black areas in Figure 2). All six selected environmental variables (Avg SST, Max SST, Min SST, Chla, Currents and Salinity) were assessed for collinearity. Then they were standardized using ranges to scale the data [21], allowing an equal contribution of the variables to the classification analysis.

The classification approach selected for this research, Self-Organizing Map (SOM), is a flexible, unsupervised neural network for data analysis and clustering [22,23,24]. Several performance studies have illustrated the advantages of SOM over other clustering methods, such as k-means, fuzzy k-mean and ISODATA [25,26]. SOM is more appropriate for large, nonlinear data sets with high dimensionality [25,26], and draws great attention in geographic information science [27] for spatial and temporal modeling and analysis [25,28,29,30,31,32,33]. In the field of marine science, there are few examples in the literature of SOM applications for the extraction of spatial patterns and classification of environmental regions. There are relatively few studies using SOM in marine environments but include (for example) the Atlantic Ocean [34] and Caribbean Sea regions [5].

SOM requires users to predefine the desired number of clusters (neurons) and the spatial arrangement of clusters (aka, neuron topology, such as linear, rectangular or square) before it runs. We produced and assessed classifications with four to 25 clusters with all possible bi-dimensional topologies (e.g., for 12 clusters 12 × 1, 3 × 4 and 4 × 3). Our goal was to identify an “optimal” number of clusters. Hexagonal grid topologies were applied in this study because they provide a better visualization of the results and smoother transitions among clusters. The neighborhood size was set to three samples and the training steps were set to 1000 iterations. Link distance was used for distance metric for its straightforward meaning and easy implementation. The full description of the algorithm and mathematical illustration can be found in [22].

Upon completion of a neural network classification of a data set into the desired number of clusters, it is necessary to validate the clusters in terms of statistical separation. There are several commonly used internal validation indices in clustering analysis, such as Silhouette Index [35], Davies-Bouldin Index [36], Calinski-Harabasz Index [37] and Dunn Index [38]. Silhouette Index (SI) provides a succinct graphical representation of how well each pixel lies within its cluster and is also used to determine the optimal number of clusters [39]. It works well with different clustering methods, such as k-means [35]. The SI is calculated using Equation (1). The scenario that maximizes the average SI determines the best partition:

(1) $$SI_{i}=\frac{b_{i}-a_{i}}{\max(a_{i},b_{i})}$$

For each pixel, *a_i_* is the average distance from the *i* th pixel to all the other pixels in the same cluster as *i* , and *b_i_* is the minimum average distance from the *i* th pixel to all pixels in a different cluster. After all *SI_i_* values have been calculated, the average SI for all clusters is calculated for each scenario. SI ranges between −1 and 1 and a clustering is valid when SI is greater than 0. With SI values close to 1, it signals a better clustering (with compact classes, well separated from the rest). Validation indices do not only identify the optimal number of clusters, but also the optimal spatial arrangement of clusters (topology of neurons).

## 3. Results

**Figure 3:**
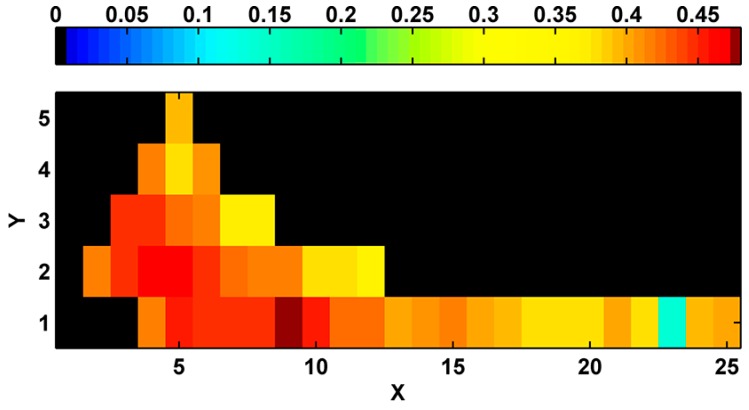
Silhouette index used to identify the best classification scenario. X and Y denote neuron arrangements in the two dimensions. Therefore, the number of classes equals to X × Y.

The best classification of biophysical marine environments from the total 43 scenarios considered was selected by comparing the goodness of the clustering structure via SI. The best scenario of classification was found with 9 clusters and linear topology (Figure 3).

**Figure 4:**
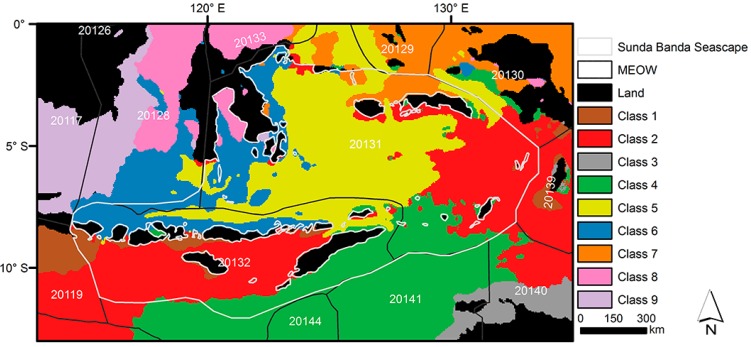
Biophysical region classification of the Sunda Banda Seascape with 9 classes. MEOW denotes Marine Ecoregions of the World [16]. Five-digit codes refer to marine ecoregions: 20126—Palawan/North Borneo; 20117—Sunda Shelf/Java Sea; 20119—Southern Java; 20128—Sulawesi Sea/Makassar Strait; 20133—Northeast Sulawesi; 20131—Banda Sea; 20132—Lesser Sunda; 20144—Exmouth to Broome; 20129—Halmahera; 20141—Bonaparte Coast; 20130—Papua; 20139—Arafura Sea; 20140—Arnhem Coast to Gulf of Carpenteria.

The selected scenario classified the biophysical environments of the study area into 9 clusters (Figure 4), where each cluster indicates a distinctive environmental region. One of these clusters (*i.e*., Class 3) was in the bounding box of study area, but not within the SBS. Although no explicit geographic information was provided when training the SOM, the classification procedure produced clusters with well-defined boundaries.

The number of pixels of each class was not evenly distributed in the SBS. Class 2, Class 4, and Class 5 included the largest number of pixels; and Class 3 was totally outside the SBS. The average and standard deviation for each biophysical variable in each class are listed in Table 1.

**Table 1:** Percentage area covered by each of the 9 classes; Average and standard deviation of 6 biophysical variables for the 9 Classes.

Class	SBS (%)	Avg SST (°C)	Max SST (°C)	Min SST (°C)	Chla (mg/m^3^)	Currents (m/s)	Salinity (PSU)
1	2.40	26.61 ± 0.70	28.79 ± 0.83	24.78 ± 0.64	1.33 ± 1.84	0.16 ± 0.11	34.04 ± 0.26
2	32.30	27.68 ± 0.29	29.49 ± 0.24	25.81 ± 0.32	0.36 ± 0.29	0.19 ± 0.06	34.23 ± 0.13
3	0	27.23 ± 0.52	30.50 ± 0.48	25.15 ± 0.50	1.54 ± 1.25	0.06 ± 0.05	34.59 ± 0.15
4	13.30	28.06 ± 0.21	30.18 ± 0.30	26.37 ± 0.21	0.33 ± 0.44	0.14 ± 0.05	34.42 ± 0.13
5	34.80	28.23 ± 0.20	29.59 ± 0.20	26.74 ± 0.37	0.23 ± 0.10	0.33 ± 0.06	33.99 ± 0.18
6	12.90	28.29 ± 0.25	29.59 ± 0.23	27.23 ± 0.33	0.31 ± 0.45	0.19 ± 0.09	33.50 ± 0.25
7	2.90	28.79 ± 0.36	29.40 ± 0.26	27.94 ± 0.53	0.48 ± 0.89	0.19 ± 0.09	34.24 ± 0.12
8	1.30	29.06 ± 0.35	29.81 ± 0.32	28.42 ± 0.40	0.41 ± 0.92	0.20 ± 0.08	33.51 ± 0.21
9	0.10	28.55 ± 0.23	29.65 ± 0.23	27.71 ± 0.34	1.40 ± 2.89	0.11 ± 0.07	32.54 ± 0.30
Range	(0, 34.8)	(26.61, 29.06)	(28.79, 30.50)	(24.78, 28.42)	(0.23, 1.54)	(0.06, 0.33)	(32.54, 34.59)

**Figure 5:**
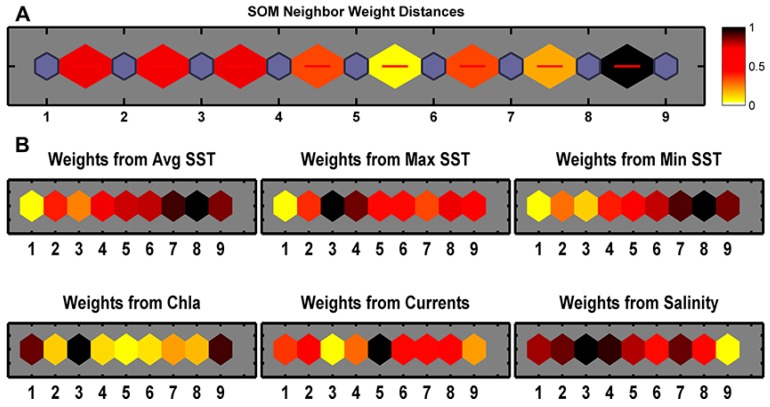
SOM topology showing the distances between neighbors and the input weights. (**A**) SOM Neighbor Weight Distance. The blue hexagons represent the classes; the red lines connect neighboring classes; the colors in the regions containing the red lines indicate the distances between classes, where the darker colors represent larger distances (more differences) and the lighter colors represent smaller distances (less differences); (**B**) Weight from each input biophysical variables. Lighter and darker colors represent smaller and larger weights, respectively.

Figure 5A shows the distinctiveness of the nine classes. The darker color indicates greater difference, such as Class 8 and Class 9, while the lighter color shows less difference, such as Class 5 and Class 6, which are relatively similar. Figure 5B shows how each biophysical variable contributes to each class, where darker colors indicates greater and lighter ones indicates less contribution. None of the weight patterns of input variables are very similar to one another, reconfirming these variables are not correlated. Based on Figure 5, the biophysical classes of the SBS are further characterized as in Table 2. Each class has a unique suite of biophysical conditions, and often aligns with established landmarks.

**Table 2:** Biophysical features and geographical regions of all classes.

Class	Biophysical Features	Geographical Regions
1	lowest temperature and high chlorophyll a	south side of Bali, Sumbawa, Fores and Sumba
2	moderate biophysical conditions	Savu Sea (in the west) and Arafura Sea (in the east)
3	high chlorophyll a and high salinity	Van Dieman Gulf and Beagle Gulf (out of the SBS)
4	low chlorophyll a and high salinity	Timor Sea
5	the lowest chlorophyll a and the highest currents	Banda Sea, Molucca Sea (to the north), Ceram Sea (to the east), Flores Sea (to the southwest).
6	low chlorophyll a and medium currents	Bali Sea, Flores Sea and Gulf of Boni
7	the second highest overall temperature	Halmahera Sea and Molucca Sea
8	highest overall temperature	Gulf of Tomini and Makassar Strait (northeast of the SBS, south to the Equator)
9	high chlorophyll a and low salinity	Java Sea

## 4. Discussion and Conclusions

Our work is the first-ever delineation of the SBS environments, and it classified the region into eight distinctive biophysical classes. Each of them represents a unique systematic combination of biophysical conditions. Classifying the marine environments into meaningful and manageable regions is often the initial step to prioritize marine conservation areas and assist resources management and spatial planning [40]. Additionally, our delineation of the biophysical environments of the SBS supplements other prevailing marine management schemes, such as the marine ecoregions of the world (MEOW) [16]. MEOW is defined on experts’ opinions and globally comparable on a biogeographic basis, for example floral and faunal composition, but it is targeted at the world’s coastal and shelf areas. Instead of only two ecoregions (*i.e*., 20131 and 20132) delineating the study area by MEOW, our biophysical classification enables comparisons among regional patterns and processes and provides more detail at the regional level (Figure 4). The eight distinctive classes should better explain patterns of biodiversity and organismal distribution. Importantly, smaller regions should be more suitable for conservation practice. With comprehensive coverage, the classification result can serve many research and conservation requirements in the area. The datasets we employed here are globally available so the systematic approach applied here can be replicated in the whole Coral Triangle using the same or other remote sensing derived and model-assimilated environmental variables.

Within the eight classes, Class 2 (32.30%), Class 4 (13.30%), Class 5 (34.80%) and Class 6 (12.90%) made up over 93% of the area in the SBS. Among these four major classes, Class 2 experiences the coolest conditions, though the differences in temperatures (Avg SST, Max SST and Min SST) are not great. The ranges of Avg SST, Max SST and Min SST among these four classes are 0.61 °C, 0.69 °C, and 1.42 °C (the overall ranges of these three variables within the study area are 2.45 °C, 1.71 °C, and 3.64 °C, respectively). These four major classes have relative low chlorophyll a, whose chlorophyll a concentration range from 0.23 mg/m^3^ to 0.36 mg/m^3^ (compared to the overall range from 0.23 mg/m^3^ to 1.54 mg/m^3^), although Class 2 has the highest upwelling level of chlorophyll a concentration (relative to the other three classes). In fact, Class 5 has the lowest chlorophyll a concentration among all classes. These four major classes are also characterized as medium-to-high currents, whose currents range from 0.14 m/s to 0.33 m/s (compared to the overall range from 0.06 m/s to 0.33 m/s), expect for Class 5, which ranks the highest in currents. There is not much similarity in terms of salinity for the four major classes; in fact, Class 5 shows the highest salinity.

Class 2 is the second largest class of the SBS. It is the only class characterized by moderate overall biophysical conditions; and the only biophysical class that divided into two major areas—*i.e*., Savu Sea in the west and Arafura Sea in the east. Featured as low chlorophyll a and high salinity, Class 4 is located in the southwest part of the SBS and aligns well with the geographical boundary of the Timor Sea. Registered as the biophysical class with highest currents and lowest chlorophyll a, Class 5 is the largest biophysical class, and mainly located in the center of the SBS—Banda Sea. Known as biophysical class with medium currents and low chlorophyll a, Class 6 is located to the west of Class 5, including areas of Bali Sea, Flores Sea and the Gulf of Boni.

By classifying the SBS into distinctive biophysical classes, this work also provides an underlying basis for more informed decisions for marine spatial planning in the SBS. With the increasing marine conservation investments in the SBS, a better understanding of the environment and ecosystems will lead to better strategic decisions, such as designating priority areas, where ecosystems are more likely to be resilient to natural and anthropogenic disturbance. During marine spatial planning and conservation practices, MPAs usually play the central role in balancing biodiversity protection with natural resource utilization [41]. MPA connectivity to other ecosystems that serve as “sources” for coral or fish larvae are more likely to be “seeded” to replenish depleted populations [42]. MPA proximity to other productive ecosystems that serve as nursery habitat, breeding grounds, and foraging grounds have been demonstrated to enhance fish abundance and diversity [43]. When designing MPAs, especially the network of MPAs, it is usually required to encompass a variety of environmental conditions to define distinct regions. Our delineation of eight classes with distinctive biophysical features and geographical locations (Table 2) in the SBS will be potentially helpful to establish the network of MPAs in this region. It can be also applied to inform strategic marine resource management in the CTI-CFF in the following three ways: (1) serve as proxies for species distribution; (2) help planning and coordinating of stratifying rapid-assessment and monitoring activities in the field in a cost-effective manner; (3) help determining areas of priority for conservation in conjunction with existing habitat data—such as areas likely to be more resilient to climate change and coral reef bleaching [44,45]. It is one of the major conservation goals of the SBS to designate the network of MPAs with the lowest trade off among biodiversity conservation and fisheries benefits. And our work contributes to this broad goal in a timely manner.

To summarize, our approach for the delineation of biophysical marine environments of the SBS not only fills a gap by bringing comprehensive data into this region, but also facilitates planning of marine conservation activities. As the first-ever work of this kind in the Coral Triangle, our delineation contributes to the framework for coastal and marine spatial planning, which will inform research and conservation practices in the Coral Triangle.

In the future, a full sensitivity analysis of the SOM parameterization will be performed to increase the accuracy of this study. While outside the scope of this paper, future work could include evaluating long term in-situ datasets in the study area with other contemporary data-driven modeling techniques, such as in [46,47,48,49]. Moreover, the input variables used in this study are relevant for other areas, both for benthic and pelagic environments. Furthermore, all the datasets used are global in scope and freely available which means that the approach is directly transferable to other parts of the world. Although the method could be applied at a global scale, computational limitations may prevent a global analysis at this stage.
